# Synthesis and antifungal activity of novel pyrazolecarboxamide derivatives containing a hydrazone moiety

**DOI:** 10.1186/1752-153X-6-51

**Published:** 2012-05-30

**Authors:** Jian Wu, Jian Wang, Deyu Hu, Ming He, Linhong Jin, Baoan Song

**Affiliations:** 1 State Key Laboratory Breeding Base of Green Pesticide and Agricultural Bioengineering, Key Laboratory of Green Pesticide and Agricultural Bioengineering, Ministry of Education, Guizhou University, Guiyang, China; 2 Research and Development Center for Fine Chemicals, Guizhou University, Guiyang, 550025, China

## Abstract

**Background:**

The plant pathogenic fungus (such as *Gibberella zeae*, *Fusarium oxysporum* and *Cytospora mandshurica*) causes devastating disease in agriculture. The pathogenic fungus is responsible for billions of dollars in economic losses worldwide each year. In order to discover new fungicidal molecule with good fungicidal activity against *G. zeae*, *F. oxysporum*, and *C. mandshurica*, we sought to combine the active sub-structure of hydrazone and pyrazole amide derivatives together to design and synthesize novel pyrazole amide derivatives containing a hydrazone moiety.

**Results:**

A series of novel pyrazole amide derivatives bearing hydrazone moiety were synthesized. Their structures were characterized by ^1^ H-NMR, ^13^ C-NMR, IR, and elemental analysis. The preliminary biological assays revealed that most of the synthesized compounds exhibit favorable antifungal activities against *G. zeae*. The activity of compounds 7a, 7f, 7g, 7h, 7i, 7j, 7l and 7q were 40.82%, 47.78%, 50.32%, 40.82%, 49.05%, 48.73%, 40.19% and 45.89%, respectively, and the synthesized compounds showed certain antifungal activities against *F. oxysporum* and *C.mandshurica*.

**Conclusion:**

A practical synthetic route to pyrazole amide derivatives containing a hydrazone moiety were synthesized by the condensation of intermediates 5-chloro-*N*-(4-subsititued-2-(hydrazinecarbonyl)-6-methylphenyl)-1,3-dimethyl-1 *H*-pyrazole-4-carboxamide with different aldehydes or ketones in ethanol at room temperature is presented, the results of the study suggested that the pyrazole amide derivatives containing hydrazone moieties could inhibit the growth of *G. zeae*, *F. oxysporium* and *C. mandshurica* to a certain extent.

## Background

The phytopathogenic fungus such as *Gibberella zeae*, which is also known as anamorph *Fusarium graminearum*, poses serious threats to agriculture. It is a broad host range pathogen that infects many crop plants, including wheat and barley, and causes head blight and rot diseases throughout the world [1]. The high incidence of plant mortality and the lack of effective control methods make the pathogen are responsible for billions of dollars in economic losses worldwide each year [2]. In recent years, because of continued moist weather during the crop growing season and the failure of chemical control in some areas due to fungicides resistance in the pathogen population, Fusarium head blight (FHB) have been endemic in the wheat-producing areas of many province of China, which have caused an estimated 30 to 50% of reduction and even completely failure of harvests in many wheat-producing areas. Therefore, the design of new compounds to deal with *G. zeae* has become one of the most important areas for fungicide research today.

**Figure 1:**
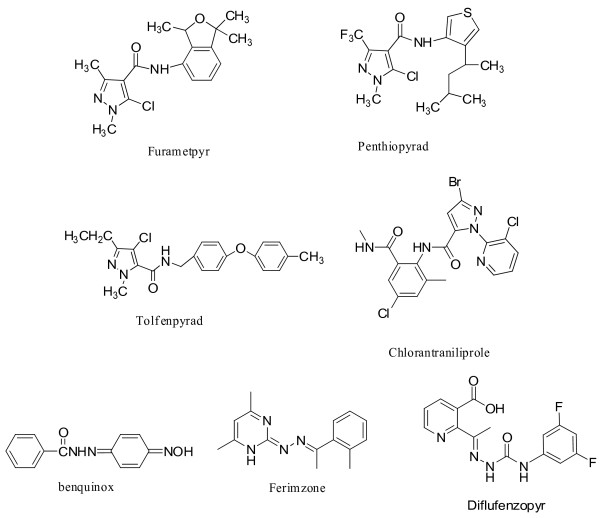
The commercialised pesticide of pyrazole amide and hydrazone derivatives.

Pyrazole amide derivatives play an important role in development of medicine and pesticide due to their broad spectrum of biological activity [3-8]. Considerable attention for the study of synthesis and biological activity of pyrazole amide derivatives has been paid in recent years [9]. Currently, some pyrazole amide derivatives have been developed and commercialized as pesticide. As shown in Figure 1, furametpyr, penthiopyrad, tolfenpyrad, and chlorantraniliprole are known for their ability to protect certain plants from severe diseases and pests. In the past few years, hydrazone derivatives has been also attracted more and more attention due to their particular physical, chemical, and biological activities [10]. Hydrazone, a class of important substructure, can be found in numerous pharmaceutically active compounds [10,11], and have been demonstrated to bear important biological activities (such as antibacterial activity [12,13], analgesic activity [14], antinociceptive activity [15], insecticidal activity [16,17], antimalarial activity [18], and antimicrobial activity [19,20]). Some of the compounds containing hydrazone substructure have been commercialized as pesticides (such as benquinox, diflufenzopyr, and ferimzone) (Figure 1). In our recent publications [21,22], several hydrazone derivatives have been synthesized and tested for their insecticidal activity and antibacterial activity, some of the hydrazone derivatives exhibited notable insecticidal activity against *Plutella xylostellaHelicoverpa armigeraCulex pipiens pallensLaphygma exiguaSpodoptera lituraNilaparvata lugens* and *Rhopalosiphum maidis*[21], and some of the hydrazone derivatives containing a pyridine moiety possessed good antibacterial activity against *Ralstonia Solanacearum*[22].

Keeping this in view, in an effort to discover new molecules against *G. zeae*, *F. oxysporum* and *C. mandshurica*, we sought to combine the sub-structural units of pyrazole amide and hydrazone together to design and synthesize novel pyrazole amide derivatives containing a hydrazone substructure. Thus, 11 novel pyrazolecarboxamide derivatives were synthesized using 3-dimethyl-1 *H*-pyrazol-5(4 *H*)-one and 2-amino-5-chloro-3-methylbenzoic acid as starting materials. All synthesized compounds were unequivocally characterized by IR, NMR and elemental analysis. The fungicidal activity on *G. zeae*, *F. oxysporium* and *C. mandshurica* were evaluated, the results showed that most of the synthesized compounds exhibit favorable antifungal activity against *G. zeae* and a certain antifungal activity against *F. oxysporum* and *C. mandshurica*, of which, compounds 7g and 7i display good activities at 50 mg/L. To the best of our knowledge, this is the first report on the antifungal activity of hydrazone derivatives containing a pyrazole moiety.

## Results and discussion

### Synthesis

**Scheme 1:**
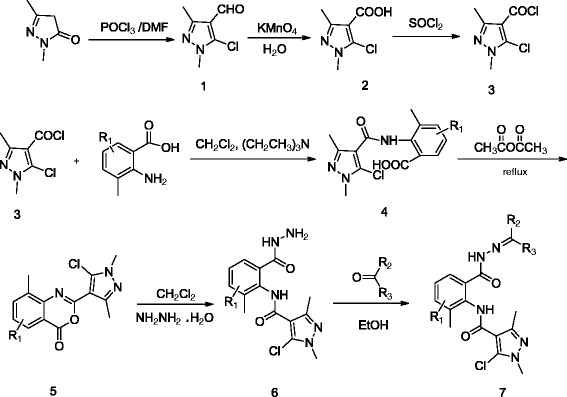
Synthetic route to target compounds 7a-7s.

The synthetic route to the title compounds is outlined in Scheme 1 [see Additional file 1. Intermediates **4** were prepared using 1,3-dimethyl-1 *H*-pyrazol-5(4 *H*)-one as starting materials. 1,3-dimethyl-1 *H*-pyrazol-5(4 *H*)-one was firstly subjected to Vilsmeier-Haack chloroformylation using *N,N*-dimethylformamide (DMF) and phosphorus oxychloride (POCl_3_) to yield 5-chloro-1,3-dimethyl-1 *H*-pyrazole-4-carbaldehyde **1**[9], which was further oxidized by potassium permanganate and following chlorinated with thionyl chloride (SOCl_2_) to provide the intermediates **3**, then intermediates **4** were prepared by treating 5-chloro-1,3-dimethyl-1 *H*-pyrazole-4-carbonyl chloride with 2-amino-3-methylbenzoic acid or 5-chloro-2-amino-3-methylbenzoic acid in CH_2_Cl_2_ in present of triethylamine in good yields, 2-(5-chloro-1,3-dimethyl-1 *H*-pyrazol-4-yl)-8-methyl-4 *H*-benzo*d*[1,3]oxazin-4-one **5** can be easily synthesized by reaction of acetic anhydride with **4** in excellent yield [21], however, it also can be prepared in a single step by the reaction of **3** with substituted 2-amino-3-methylbenzoic acid as describing in the literature [21,22]. Finally, compounds **6** were conveniently obtained with excellent yield (>90%) by treatment of **5** with 80% hydrazine hydrate, subsequent treatment of **6** with different ketones and aldehydes (or hemiacetals) in ethanol at room temperature afforded the desired compounds (**7a** to **7s**) with excellent yields. The structures of all new compounds were confirmed by their spectra (IR, ^1^ H NMR, ^13^ C NMR) and elemental analytical data. Additional file shows the structures, yields and elemental analysis data for title compounds in more detail [see Additional file 2. Moreover, hydrazone derivatives have two configurations due to the existence of double bond (C = N), for the title compounds, *E* and *Z* configuration can be observed in the ^1^ H-NMR spectra, and the *E* isomer was found predominantly, and the ratio between *E* and *Z* configuration can be calculated based on the integral area of *E* and *Z* isomers in ^1^ H-NMR spectra. Take compound **7k** as an example, the *E* isomer of -CON*H*N proton can be found at *δ* 9.59, and the proton of *Z* isomer was appeared at *δ* 9.08, and the ratio of *E* isomer and *Z* isomer for **7k** is 3.43, approximately.

### Biological activity and structure-activity relationship

All of the synthesized compounds 7a-7s was evaluated *in vitro* against three pathogenic fungi, *G. zeaeF. oxysporium*, and *C. mandshurica* using the poison plate technique [23]. Carbendazim, one of the commercial fungicides for controlling *G. zeaeF. oxysporium*, and *C. mandshurica* was used as positive control. The results provided in Table 1 indicated that most of the prepared compounds had weak to good antifungal activity against the tested fungi at 50 mg/L. Compounds 7a, 7f, 7g, 7h, 7i, 7j, 7l and 7q showed 40.82%, 47.78%, 50.32%, 40.82%, 49.05%, 48.73%, 40.19% and 45.89% activity against *G. zeae*, respectively. However, most of the synthesized compounds displayed lower activities against *F. oxysporium* and *C. mandshurica*, only compounds **7d** and **7i** showed 33.23%, 35.76% activity against *F. oxysporum* at 50 mg/L, respectively; compounds **7i** possessed 37.25% activity against *C. mandshurica* at 50 mg/L. Structure activity relationship (SAR) based on activity against *G. zeae* showed that the compound containing two substructures of 5-chloro-1,3-dimethyl-1 *H*-pyrazole (compound 7g) is proven to be more active than other compounds; and the compound containing a furan moiety (7i) also display good activity, which is very close to that of compound 7g. We can conclude that changing the substituent on benzene could lead to a remarkable change in activity, for instance, the compounds without any substituent on benzene (7f, 7l) displayed much higher activities than the compounds with chorine at the 4-position of benzene (7m, 7r). Furthermore, the compounds with the same substituent but at different position on phenyl ring exhibited different activity. For instance, the activity of the compound 7 l which with 4-chloro substituent on phenyl ring was 40.19%, but the activity of the compound **7r** which with 3-chloro substituent on phenyl ring was 6.65%; Moreover, different type of substituent on the phenyl ring (in R_2_ group) also affect the activity of the compounds, such as the compound 7q with methyl substituting at 4-position on phenyl ring possessed good activity on *G. zeae*, and the compound 7p with fluorine substituting at 2-position on phenyl ring displayed moderate activity, while the compound **7r** with chlorine substituting at 3-position on phenyl ring demonstrate weak activity against *G. zeae*.

**Table 1:** Antifungal activity of the title compounds 7a-7s at 50 mg/L

**Compound**	**Inhibition rate**^**a**^**(%)**		
	***G. zeae***	***F. oxysporum***	***C. mandshurica***
**7a**	40.82 ± 0.88	8.05 ± 1.61	16.11 ± 1.09
**7b**	39.87 ± 0.92	10.40 ± 3.34	15.44 ± 1.54
**7c**	31.96 ± 1.59	12.75 ± 1.48	12.75 ± 1.39
**7d**	33.23 ± 1.0	33.22 ± 1.99	16.78 ± 1.43
**7e**	35.76 ± 2.32	34.56 ± 3.11	19.46 ± 1.58
**7f**	47.78 ± 1.54	10.07 ± 2.92	12.75 ± 1.39
**7g**	50.32 ± 2.57	8.72 ± 1.45	7.38 ± 1.19
**7h**	40.82 ± 0.88	5.03 ± 1.22	16.11 ± 1.21
**7i**	49.05 ± 3.02	27.85 ± 1.62	37.25 ± 1.40
**7j**	48.73 ± 2.74	11.41 ± 1.32	20.13 ± 1.24
**7k**	37.66 ± 1.78	15.10 ± 2.03	8.39 ± 1.17
**7l**	40.19 ± 2.05	8.39 ± 1.54	16.11 ± 1.21
**7m**	29.11 ± 1.38	13.42 ± 1.43	12.08 ± 1.18
**7n**	36.39 ± 2.64	14.09 ± 1.40	15.10 ± 1.57
**7o**	36.08 ± 1.58	5.03 ± 1.40	14.09 ± 1.20
**7p**	37.34 ± 1.13	11.07 ± 1.45	11.07 ± 1.26
**7q**	45.89 ± 3.91	9.73 ± 1.25	16.44 ± 1.06
**7r**	6.65 ± 2.98	10.40 ± 1.47	22.48 ± 1.68
**7s**	39.24 ± 1.43	5.03 ± 1.31	11.07 ± 1.15
Carbendazim^b^	100.00 ± 3.74	100 ± 10.90	100 ± 8.76

^a^Average of three replicates, ^b^The commercial agricultural fungicide carbendazim was used for the comparison of activity.

### Experimental

#### Chemistry

Unless otherwise stated, all the reagents and reactants were purchased from commercial suppliers; melting points were uncorrected and determined on a XT-4 binocular microscope (Beijing Tech Instrument Co., China). The ^1^ H-NMR and ^13^ C-NMR spectra were recorded on a JEOL ECX 500 NMR spectrometer (JEOL Ltd., Japan) at room temperature operating at 500 MHz for ^1^ H-NMR and 125 MHz for ^13^ C-NMR by using CDCl_3_ or DMSO as solvents and TMS as an internal standard; infrared spectra were recorded in KBr on a IR Pristige-21 spectrometer (Shimadzu corporation, Japan); elemental analysis was performed on an Elemen tal Vario-III CHN analyzer (Elementar, German). The course of the reactions was monitored by TLC; analytical TLC was performed on silica gel GF 254. Intermediates 1, 2, 3, and 4 were prepared according to the reported methods [9] and used without further purifications, the process for preparing of them can be found in Additional file 3.

#### Antifungal biological assay

All the compounds 7a-7 s were tested for in vitro antifungal activity using the poison plate technique [23]. The antifungal activity was evaluated against three pathogenic fungi, *G. zeaeF. oxysporium*, and *C. mandshurica*, The results of preliminary bioassays were compared with the experimental data of a commercial agricultural fungicide, Carbendazim. Compounds were dissolved in dimethyl sulfoxide (1 mL) before mixing with potato dextrose agar (PDA, 90 mL). The compounds were tested at a concentration of 50 mg/L. All fungi were incubated in PDA at 27 ± 1°C for 4 days to get new mycelium for antifungal assay. Then mycelia dishes of approximately 4 mm diameter were cut from culture medium, and one of them was picked up with a sterilized inoculation needle and inoculated in the center of PDA plate aseptically. The inoculated plates were incubated at 27 ± 1°C for 5 days. DMSO in sterile distilled water served as negative control, while carbendazim acted as positive control. For each treatment, three replicates were conducted. The radial growth of the fungal colonies was measured and the data were statistically analyzed. The inhibitory effects of the test compounds in vitro on these fungi were calculated by the formula:

I (%)=[(C-T)/(C-0.4)]×100

Where C represents the diameter of fungi growth on untreated PDA, and T represents the diameter of fungi on treated PDA while I mean the inhibition rate.

## Conclusion

In summary, a novel series of pyrazole amide derivatives bearing hydrazone moieties were synthesized. The synthesized compounds were characterized by spectral data (^1^ H NMR, ^13^ C NMR, IR) and elemental analysis. All of the compounds were subjected to fungicidal activities *in vitro* against *G. zeae*, *F. oxysporium* and *C. mandshurica.* The results indicated that the synthesized compounds possessed weak to good antifungal activities against the tested fungi, among them, compounds 7f, 7g, 7i, 7j displayed good antifungal activities against *G. zeae*; 7e and 7i display moderate activities against *F. oxysporum* and *C. mandshurica* respectively. Further studies are currently underway to optimize to enhance the antifungal activity of the pyrazole amide derivatives bearing hydrazone substructure.

## Competing interests

The authors declare that they have no competing interests.

## Authors’ contributions

The current study is an outcome of constructive discussion with BAS, LHJ and DYH who offered necessary guidance to JW and JW to carry out their synthesis and characterization experiments. Both of JW and JW were also involved in the drafting of the manuscript. MH performed the Antifungal tests; LHJ carried out the ^1^ H NMR, ^13^ C NMR spectral analyses and elemental analysis. BAS were involved in revising the manuscript. All authors read and approved the final manuscript.

## Supplementary Material

Additional file 1 **Synthetic route to target compounds 7a-7s.** Synthetic sequence to pyrazole amide derivatives containing a hydrazone moiety from 6. Click here for file

Additional file 2 **Yield and elemental analyses data for title compounds 7a-s.** Which contains the table about structure, yield and elemental analyses data for title compounds 7a-s. Click here for file

Additional file 3 **Experimental details and data of title compounds 7a-s.** Which includes the experimental procedure, spectroscopic data of intermediate 5, intermediate 6, title compounds 7a-s, copies of ^1^H NMR and ^13^C NMR. Click here for file
