# Supplementary material for: Synthesis and antifungal activity of novel pyrazolecarboxamide derivatives containing a hydrazone moiety
Source: Chem Cent J. 2012 May 30;6:51. doi: 10.1186/1752-153X-6-51 (PMC3539435; doi:10.1186/1752-153X-6-51)
Supplement: Additional file 1 — Synthetic route to target compounds 7a-7s. Synthetic sequence to pyrazole amide derivatives containing a hydrazone moiety from 6. [file 1752-153X-6-51-S1.doc]

Additional file 1

**Synthesis and Antifungal Activity of a Novel of Pyrazolecarboxamide Derivatives Bearing Hydrazone Moiety**

Jian Wu, Jian Wang, Deyu Hu, Ming He, Linhong Jin, Baoan Song*

State Key Laboratory Breeding Base of Green Pesticide and Agricultural Bioengineering, Key Laboratory of Green Pesticide and Agricultural Bioengineering, Ministry of Education, Guizhou University, Guiyang 550025, China.

Author to whom correspondence should be addressed;

Tel.: +86 851 362 0521; Fax: +86 851 362 2211.

E-Mail: JW: jianwu2691@yahoo.com.cn

JW: wangjmy2008@126.com

DYH: fcc.dyhu@gzu.edu.cn

MH: michael.lee1983@yahoo.com.cn

LHJ: bhadury@gzu.edu.cn

BAS: basong@gzu.edu.cn

**Scheme 1** Synthesis of pyrazolecarboxamide derivatives bearing Hydrazone moiety 7
