# Supplementary material for: Synthesis and antifungal activity of novel pyrazolecarboxamide derivatives containing a hydrazone moiety
Source: Chem Cent J. 2012 May 30;6:51. doi: 10.1186/1752-153X-6-51 (PMC3539435; doi:10.1186/1752-153X-6-51)
Supplement: Additional file 2 — Yield and elemental analyses data for title compounds 7a-s. Which contains the table about structure, yield and elemental analyses data for title compounds 7a-s. [file 1752-153X-6-51-S2.doc]

**Synthesis and Antifungal Activity of a Novel of Pyrazolecarboxamide Derivatives Bearing Hydrazone Moiety**

Jian Wu, Jian Wang, Deyu Hu, Ming He, Linhong Jin, Baoan Song*

State Key Laboratory Breeding Base of Green Pesticide and Agricultural Bioengineering, Key Laboratory of Green Pesticide and Agricultural Bioengineering, Ministry of Education, Guizhou University, Guiyang 550025, China.

Author to whom correspondence should be addressed;

Tel.: +86 851 362 0521; Fax: +86 851 362 2211.

E-Mail: JW: jianwu2691@yahoo.com.cn

JW: wangjmy2008@126.com

DYH: fcc.dyhu@gzu.edu.cn

MH: michael.lee1983@yahoo.com.cn

LHJ: bhadury@gzu.edu.cn

BAS: basong@gzu.edu.cn

**Table 2. Structure, yield and elemental analysis data for title compounds 7a-s**

|  |  |  |  |  | Elemental Analysis (Calcd./Found) | | |
| --- | --- | --- | --- | --- | --- | --- | --- |
| NO. | R_1_ | R_2_ | R_3_ | Yield (%) | C | H | N |
| **7a** | H | CHCH_2_CH_3_ | H | 80 | 56.43/56.64 | 5.57/5.54 | 19.36/19.59 |
| **7b** | H | CHCH_2_CH_2_CH_3_ | H | 79 | 57.52/57.50 | 5.90/6.27 | 18.63/18.92 |
| **7c** | H | CH_3_ | CH_3_ | 81 | 56.43/56.57 | 5.57/5.86 | 19.36/19.49 |
| **7d** | H | quinolone-2-yl | H | 77 | 62.54/62.23 | 4.59/4.35 | 18.23/18.01 |
| **7e** | H | pyridin-2-yl | H | 75 | 58.47/58.21 | 4.66/4.44 | 20.45/20.32 |
| **7f** | H | N(CH_3_)_2_ | H | 85 | 54.18/54.53 | 5.62/5.55 | 22.30/22.11 |
| **7g** | H | 5-chloro-1,3-dimethyl-1H-pyrazol-4-yl | H | 86 | 51.96/51.57 | 4.58/4.23 | 21.21/21.19 |
| **7h** | H | 5-chloro-3-  methyl-1-phenyl-1H-pyrazol-4-yl | H | 84 | 57.26/56.89 | 4.42/4.67 | 18.70/18.38 |
| **7i** | H | furan-2-yl | H | 78 | 57.07/57.30 | 4.54/4.82 | 17.52/17.64 |
| **7j** | H | CHCH(CH_3_)_2_ | H | 80 | 57.52/57.02 | 5.90/5.59 | 18.63/18.30 |
| **7k** | 4-Cl | 1-methyl-1H-  pyrrol-2-yl | H | 73 | 53.70/53.24 | 4.51/4.62 | 18.79/18.64 |
| **7l** | H | 4-chlorophenyl | H | 88 | 56.77/56.34 | 4.31/4.51 | 15.76/15.58 |
| **7m** | 4-Cl | N(CH_3_)_2_ | H | 89 | 49.64/49.21 | 4.90/4.61 | 20.43/20.14 |
| **7n** | 4-Cl | pyridin-2-yl | H | 72 | 53.94/53.58 | 4.07/4.19 | 18.87/18.71 |
| **7o** | 4-Cl | quinolone-2-yl | H | 74 | 57.56/57.95 | 4.07/4.16 | 16.97/16.89 |
| **7p** | 4-Cl | 2-fluorophenyl | H | 84 | 54.56/54.41 | 3.92/3.69 | 15.15/15.24 |
| **7q** | 4-Cl | 4-methylphenyl | H | 83 | 57.65/57.16 | 4.62/4.50 | 15.28/15.25 |
| **7r** | 4-Cl | 3-chlorophenyl | H | 81 | 52.68/52.42 | 3.79/3.85 | 14.63/14.39 |
| **7s** | 4-Cl | 4-chloro-3-nitro  phenyl | H | 71 | 48.16/48.02 | 3.27/3.59 | 16.05/15.80 |
