# Supplementary material for: Synthesis and antifungal activity of novel pyrazolecarboxamide derivatives containing a hydrazone moiety
Source: Chem Cent J. 2012 May 30;6:51. doi: 10.1186/1752-153X-6-51 (PMC3539435; doi:10.1186/1752-153X-6-51)

Additional file 3

Synthesis and antifungal activity of novel pyrazolecarboxamide derivatives containing a hydrazone moiety

Jian Wu, Jian Wang, Deyu Hu, Ming He, Linhong Jin, Baoan Song*

State Key Laboratory Breeding Base of Green Pesticide and Agricultural Bioengineering, Key Laboratory of Green Pesticide and Agricultural Bioengineering, Ministry of Education, Research and Development Center for Fine Chemicals, Guizhou University, Guiyang 550025, China.

Author to whom correspondence should be addressed;

Tel.: +86 851 362 0521; Fax: +86 851 362 2211.

E-Mail: JW: jianwu2691@yahoo.com.cn

JW: wangjmy2008@126.com

DYH: fcc.dyhu@gzu.edu.cn

MH: michael.lee1983@yahoo.com.cn

LHJ: bhadury@gzu.edu.cn

BAS: basong@gzu.edu.cn

**Supporting Information**

**Table contents**

[*Experimental Procedure* S1](#__RefHeading___Toc319170078)

[*The Data of Intermediates 5a, 5b, 6a, 6b* S2](#__RefHeading___Toc319170079)

[*The physical and spectral data for title compounds 7a-s.* S3](#__RefHeading___Toc319170080)

[*References* S13](#__RefHeading___Toc319170081)

[Copies of ^1^H-NMR and ^13^C-NMR of 7a-s S14](#__RefHeading___Toc319170082)

# Experimental Procedure

**General Synthetic Procedure for intermediate 1 [1].**

To a well stirred cold solution of DMF (60 mmol) was added dropwise phosphoryl trichloride (90 mmol). The resulting mixture was stirred at room temperature for another 20 min. To the above solution was added 1, 3-dimethyl-1H-pyrazol-5(4H) - one (30 mmol), then it was heated to 85 °C for 5 h. The reaction mixture was cooled and poured into cold water (100 ml). The pH of the mixture was adjusted to 7 by sodium hydroxide solution. The resulting solution was extracted with ethyl acetate (3 × 30 ml). The organic layer was dried over anhydrous sodium sulfate. The solvent was evaporated under reduced pressure, and then the residue was recrystallized from ethyl acetate to give a colourless crystal to be used for the next step.

**General Synthetic Procedure for intermediate 2 [2].**

To a solution of 5-chloro-1,3-dimethyl-1H-pyrazole-4-carbaldehyde **1** (10 mmol)and sodium carbonate (10 mmol) in water (50 mL), potassium permanganate solution was added dropwise through a dropping funnel. After stirring for 30 min at room temperature, the mixture was vigorously stirred and maintained at 60 °C for 1 h. Excess permanganate were destroyed by addition of sodium metabisulfite. The mixture was then adjusted to strongly alkaline with sodium hydroxide solution, the insoluble part was removed through filtration, and the filtrate was acidified by concentrated hydrochloric acid. After the solvent had been removed under reduced pressure, 5-chloro-l, 3-dimethylpyrazole-4-carboxylic acid **2** was obtained as a white powder. Crystallization from aqueous acetic acid gave the product as colourless cubic crystals.

**General Synthetic Procedure for intermediate 3 [3].**

A Three-necked 50 mL round-bottomed flask fitted with a thermometer, and a magnetic stir bar was charged with 5-chloro-1,3-dimethyl-1H-pyrazole-4-carboxylic acid **2**, and then added 20 mL of thionyl chloride. The resulting mixture was refluxed for 6-8 h, and evaporated in vacuo to obtain the crude acid chloride. Then the residue was recrystallized from dried Petroleum ether to give a white crystal to be used for the next step.

**General Synthetic Procedure for intermediate 4 [4].**

The solution of 5-chloro-1, 3-dimethyl-1H-pyrazole-4-carbonyl chloride **3** (10 mmol) in dichloromethane (10 mL) was added slowly to a stirred solution of 2-amino-3- methylbenzoic acid (10 mmol) in dichloromethane (100 mL) at 0 °C. After 5 min, triethylamine (15 mmol) was added dropwise. The solution was warmed to room temperature and stirred for 10 h, diluted with dichloromethane (20 mL), and washed with 1 mol/L aq. HCl solution, water, and brine. The organic extract was separated, dried (MgSO4), filtered, and concentrated. The residue was purified by silica-gel column eluted with ethyl methanol/chloroform (volume ratio 1: 12) and the pure fractions were combined and evaporated to obtain the white product.

**General Synthetic Procedure for intermediate 5**

To a 100 mL round-bottomed flask were placed 2-(5-chloro-1, 3-dimethyl-1H- pyrazole-4-carboxamido)-3-methylbenzoic acid **4** (10 mmol), and added 20 mL of acetic anhydride, then the mixture was refluxed for 6 h, the mixture was cooled, filtered and washed with saturated sodium carbonate solutionthe (3× 10mL). After the solvent had been removed under reduced pressure, the residue was recrystallized from ethanol as white crystals.Using this method, the compounds **5** could be obtained at 83% yield.

**General Synthetic Procedure for intermediate 6**

The 2-(5-chloro-1,3-dimethyl-1H-pyrazol-4-yl)-8-methyl-4H-benzo[d][1,3]oxazin-4- one **5** (10 mmol) was dissolved in dichloromethane (12 mL) stirred at room temperature, then the 80% hydrazine hydrate (15 mmol) was added dropwise, and the resultant reaction mixture was stirred for 4 h at room temperature. The resulting solid was collected by vacuum filtration, and the residue was recrystallized from ethanol as white crystals.

**General procedure for the synthesis of 7a-s**

The substituted aldehydes (0.5 mmol) were added to a solution of intermediate **6** (0.5 mmol) in ethanol at room temperature. Completion of the reaction was checked by TLC. After the completion of the reaction, the mixture was collected by vacuum filtration and recrystallized from ethanol.

# The Data of Intermediates 5a, 5b, 6a, 6b

**Data for 2-(5-chloro-1, 3-dimethyl-1*H*-pyrazol-4-yl)-8-methyl-4*H*-benzo[*d*] [1,3] oxazin-4-one (5a)**

White crystal, M.p. 202-204 °C; ^1^H NMR (500 MHz, CDCl_3_, ppm): *δ* 8.00 (d, *J* = 8.00 Hz, 1H, 6-Ph-H), 7.63 (d, *J* = 7.45 Hz, 1H, 7-Ph-H), 7.35 (t, *J* = 7.45 Hz, 1H, 5-Ph-H), 3.87 (s, 3H, NCH_3_), 2.62 (s, 3H, pyrazole-CH_3_), 2.58 (s, 3H, Ph-CH_3_).

**Data for 6-chloro-2-(5-chloro-1, 3-dimethyl-1*H* -pyrazol-4-yl) -8-methyl-4*H* -benzo [*d*] [1, 3] oxazin-4-one** **(5a)**

White crystal, M.p. 224-226 °C; ^1^H NMR (500 MHz, CDCl_3_, ppm): *δ* 7.99 (s, 1H, 5-Ph-H), 7.63 (s, 1H, 7-Ph-H), 3.86 (s, 3H, NCH_3_), 2.60 (s, 3H, pyrazole-CH_3_), 2.55 (s, 3H, Ph-CH_3_).

**Data for** **5-chloro-*N*-(2-(hydrazinecarbonyl)-6-methylphenyl)-1, 3-dimethyl -1*H*- pyrazole-4-carboxamide** **(6a)**

White crystal, M.p. 239-241 °C; ^1^H NMR (500 Hz, DMSO-*d_6_*, ppm): *δ* 9.88 (s, 1H, -CON*H*Ar), 9.71 (s, 1H, -CON*H*NH_2_)，7.40 (t, 2H, *J* = 8.05 Hz, 4,6-Ph-H ), 7.25 (t, 1H, *J* = 7.70 Hz, 5-Ph-H ), 4.50(s, 2H, -CONHN*H*_2_ ), 3.79(s, 3H, NCH_3_), 2.37 (s, 3H, pyrazole-CH_3_), 2.21 (s, 3H, Ph-CH_3_).

**Data for** **5-chloro-*N*-(4-chloro-2-(hydrazinecarbonyl)-6-methylphenyl)-1, 3- dimethyl-1*H*-pyrazole-4-carboxamide (6b)**

White crystal, M.p. 247-249 °C; ^1^H NMR (500 Hz, DMSO-*d_6_*, ppm): *δ* 9.78 (s, 1H, -CON*H*Ar), 9.75 (s, 1H, -CON*H*NH_2_)，7.50 (d, 2H, *J* = 2.30 Hz, 6-Ph-H ), 7.40 (d, 1H, *J* = 2.30 Hz, 4-Ph-H ), 4.49(s, 2H, -CONHN*H*_2_ ), 3.79(s, 3H, NCH_3_), 2.35 (s, 3H, pyrazole-CH_3_), 2.21 (s, 3H, Ph-CH_3_).

# The physical and spectral data for title compounds 7a-s.

**5-chloro-1, 3-dimethyl-*N*-(2-methyl-6-(2-propylidenehydrazinecarbonyl)phenyl)- 1*H*- pyrazole-4-carboxamide (7a)**

White solid, M.p. 246~248 °C. ^1^H NMR (500 MHz, CDCl_3_, ppm): *δ* 10.04 (s, 1H, -CON*H*Ar), 9.94(s, 1H, -CON*H*N)，7.57～7.26 (m, 3H, Ar-H), 7.03 (t, *J* = 7.15 Hz, 1H, -N=*CH*), 3.85 (s, 3H, NCH_3_), 2.53 (s, 3H, pyrazole-CH_3_), 2.43～2.37 (m, 2H, -CH_2_-), 2.26 (s, 3H, Ph-CH_3_), 1.16 (t, *J* = 7.20 Hz, 3H, -CH_2_C*H*_3_); ^13^C NMR (125 MHz, CDCl_3_): *δ* 164.3, 161.5, 154.5, 150.2, 135.9, 135.3, 133.9, 128.7, 127.6, 125.9, 125.3, 111.5, 36.4, 26.1, 19.6, 14.6, 11.1. IR (KBr, cm^-1^): ν 3311.8, 3219.2, 3062.9, 2960.7, 2933.7, 2850.8, 1660.7, 1639.5, 1624.1, 1556.6, 1525.7, 1504.5, 1458.2, 1446.6, 1377.2.

***N*-(2-(2-butylidenehydrazinecarbonyl)-6-methylphenyl)-5-chloro-1, 3-dimethyl- 1*H*-pyrazole-4-carboxamide) (7b)**

White solid, M.p. 246~248 °C. ^1^H NMR (500 MHz, CDCl_3_, ppm): *δ* 9.79 (s, 1H, -CON*H*Ar), 9.71(s, 1H, -CON*H*N)，7.53 (t, *J* = 5.72 Hz, 1H, 4-Ph-H), 7.30 (d, *J* = 8.00 Hz, 2H, 6-Ph-H, =CH), 7.10 (d, *J* = 7.72 Hz, 1H, 5-Ph-H), 3.83 (s, 3H, NCH_3_), 2.51 (s, 3H, pyrazole-CH_3_), 2.372.32 (m, 2H, CH_2_), 2.28 (s, 3H, Ph-CH_3_), 1.60～1.54 (m, 2H, -CH_2_-), 0.99 (t, *J* = 7.45 Hz, 3H, -CH_2_C*H*_3_); ^13^C NMR (125 MHz, CDCl_3_): *δ* 164.4, 161.4, 153.4, 150.3, 136.1, 135.1, 133.9, 128.6, 128.1, 126.0, 125.3, 111.5, 36.4, 34.6, 20.2, 19.5, 14.6, 13.9. IR (KBr, cm^-1^): ν 3306.0, 3213.4, 3064.9, 2955.0, 2929.9, 2970.0, 1660.7, 1624.1, 1556.6, 1523.7, 1504.5, 1456.3, 1444.7, 1377.2.

**5-chloro-1, 3-dimethyl-*N*-(2-methyl-6-(2-(propan-2-ylidene)hydrazinecarbonyl) phenyl) -1*H*-pyrazole-4-carboxamide (7c)**

White solid, M.p. 232~234 °C. ^1^H NMR (500 MHz, CDCl_3_, ppm): *δ* 9.12 (s, 1H, -CON*H*Ar), 8.80(s, 1H, -CON*H*N), 7.23~7.42 (m, 3H, Ar-H) 3.83 (s, 3H, NCH_3_), 2.46 (s, 3H, pyrazole-CH_3_), 2.32 (s, 3H, Ph-CH_3_), 2.12 (s, 3H, -CC*H*_3_), 1.92 (s, 3H, -CC*H*_3_); ^13^C NMR (125 MHz, CDCl_3_): *δ* 168.2, 165.0, 160.7, 150.7, 136.4, 134.6, 133.8, 128.1, 126.6, 125.9, 125.2, 111.5, 36.4, 25.7, 19.4, 16.9, 14.6. IR (KBr, cm^-1^): ν 3356.7, 3255.5, 3186.4, 3030.2, 2960.7, 2929.9, 1661.8, 1593.2, 1537.2, 1510.3, 1469.8, 1456.3, 1429.3, 1371.4.

**5-chloro-1,3-dimethyl-*N*-(2-methyl-6-(2-(quinolin-2-ylmethylene)hydrazine carbonyl) phenyl)-1*H*-pyrazole-4-carboxamide (7d)**

White solid, M.p. 244~246 °C. ^1^H NMR (500 MHz, CDCl_3_, ppm): *δ* 10.94 (s, 1H, -CON*H*Ar), 9.61(s, 1H, -CON*H*N)，8.44,(s, 1H, -N=*CH*), 8.29 (d, 1H, *J*= 8.55 Hz 4-quinoline-H), 8.17 (d, 1H, *J*= 8.55 Hz 5-quinoline-H), 8.08 (d, 1H, *J*= 8.00 Hz 8-quinoline-H), 7.83 (d, 1H, *J*= 8.00 Hz 3-quinoline-H), 7.73 (t, 1H, *J*= 7.45 Hz 7-quinoline-H), 7.58 (t, 1H, *J*= 7.45 Hz 6-quinoline-H), 7.45 (d, 1H, *J*= 7.45 Hz 6-Ph-H), 7.31 (d, 1H, *J*= 7.45 Hz 5-Ph-H), 7.12 (d, 1H, *J*= 6.90 Hz 4-Ph-H),3.84 (s, 3H, NCH_3_), 2.55 (s, 3H, pyrazole-CH_3_), 2.32 (s, 3H, Ph-CH_3_); ^13^C NMR (125 MHz, CDCl_3_): *δ* 165.0, 161.5, 153.3, 150.7, 149.4, 148.0, 136.9, 136.5, 134.8, 134.4, 130.0, 129.5, 128.6, 128.6, 128.3, 127.8, 127.5, 126.5, 125.6, 118.6, 111.4, 36.4, 19.4, 14.7. IR (KBr, cm^-1^): ν 3385.1, 3228.8, 3057.1, 3010.9, 2929.9, 2848.9, 2740.9, 1658.8, 1643.4, 1589.3 1533.4, 1550.8, 1506.4, 1464.0, 1429.3.

**5-chloro-1,3-dimethyl-*N*-(2-methyl-6-(2-(pyridin-2-ylmethylene)hydrazinecarbonyl)phenyl)-1*H*-pyrazole-4-carboxamide (7e)**

White solid, M.p. 244~246 °C. ^1^H NMR (500 MHz, CDCl_3_, ppm): *δ* 10.89 (s, 1H, -CON*H*Ar), 9.54(s, 1H, -CON*H*N)，8.62 (d, 1H, *J*= 4.60 Hz, 6-pyridin-H ), 8.21(s, 1H, =CH), 8.17 (d, 1H, *J*= 8.05 Hz, 3-pyridin-H ), 7.74 (t, 1H, *J*= 7.45 Hz, 4-pyridin-H ), 7.41 (d, 1H, *J*= 7.45 Hz, 5-Ph-H ), 7.31 (t, 2H, *J*= 11.75 Hz, 5-pyridin-H, 5-Ph-H ), 7.11 (t, 1H, *J*= 7.73 Hz, 6-Ph-H ), 3.84 (s, 3H, NCH_3_), 2.53 (s, 3H, pyrazole-CH_3_), 2.27 (s, 3H, Ph-CH_3_); ^13^C NMR (125 MHz, CDCl_3_): *δ* 165.1, 161.4, 155.1, 153.0, 150.6, 149.3, 148.9, 136.7, 136.6, 135.0, 134.2, 128.5, 126.2, 125.7, 124.6, 121.3, 111.4, 36.4, 19.4, 14.7. IR (KBr, cm^-1^): ν 3327.2, 3205.7, 3062.9, 3055.2, 2974.2, 2929.9, 2856.6, 2740.9, 1664.6, 1643.4, 1587.4, 1552.7, 1521.8, 1494.8, 1469.8, 1431.2, 1377.2, 1334.1.

**5-chloro-*N*-(2-(2-((dimethylamino)methylene)hydrazinecarbonyl)-6-methylphenyl)-1,3-dimethyl-1*H*-pyrazole-4-carboxamide (7f)**

White solid, M.p. 247~249 °C. ^1^H NMR (500 MHz, CDCl_3_, ppm) *δ:* 9.74 (s, 1H, -CON*H*Ar), 8.76(s, 1H, -CON*H*N), 7.70(s, 1H, =CH), 7.12~7.32 (m, 3H, Ar-H) 3.82 (s, 3H, NCH_3_), 2.92 (s, 6H, N(CH_3_)_2_), 2.50 (s, 3H, pyrazole-CH_3_), 2.28 (s, 3H, Ph-CH_3_); ^13^C NMR (125 MHz, DMSO-*d_6_*): *δ* 163.4, 159.9, 155.2, 148.7, 135.8, 135.2, 132.7, 131.1, 127.7, 126.0, 125.9, 112.3, 39.5, 39.5, 36.4, 19.4, 14.4. IR (KBr, cm^-1^): ν3267.4, 3225.0, 3074.5, 2924.1, 2875.9, 1649.1, 1627.9, 1606.7, 1581.6, 1504.5, 1456.3, 1377.2.

**5-chloro-*N*-(2-(2-((5-chloro-1,3-dimethyl-1*H*-pyrazol-4-yl)methylene)hydrazinecarbonyl)-6-methylphenyl)-1,3-dimethyl-1*H*-pyrazole-4-carboxamide (7g)**

White solid, M.p. >250 °C. ^1^H NMR (500 Hz, CDCl_3_, ppm): *δ* 9.90 (s, 1H, -CON*H*Ar), 9.40 (s, 1H, -CON*H*N); 8.07 (s, 1H, =CH), 7.39~7.14 (m, 3H, Ar-H), 3.84 (s, 3H, NCH_3_); 3.79 (s, 3H, NCH_3_), 2.52 (s, 3H, pyrazole-CH_3_), 2.50 (s, 3H, pyrazole-CH_3_), 2.30 (s, 3H, Ph-CH_3_); ^13^C NMR (125 MHz, DMSO-*d_6_*): *δ* 164.1, 160.5, 148.3, 147.0, 140.8, 136.4, 134.9, 133.1, 132.1, 128.0, 127.3, 126.5, 126.3, 112.9, 111.5, 36.6, 36.4, 19.1, 14.6, 14.1. IR (KBr, cm^-1^): ν3325.3, 3217.3, 3198.0, 3064.9, 2927.9, 2873.9, 1676.1, 1635.6, 1608.6, 1568.3, 1521.8, 1500.6, 1458.2, 1377.2.

**5-chloro-*N*-(2-(2-((5-chloro-3-methyl-1-phenyl-1*H*-pyrazol-4-yl) methylene) hydrazinecarbonyl)-6-methylphenyl)-1, 3-dimethyl-1*H*-pyrazole-4-carboxamide (7h)**

White solid, M.p. >250 °C. ^1^H NMR (500 Hz, CDCl_3_, ppm): *δ* 9.96 (s, 1H, -CON*H*Ar), 9.38 (s, 1H, -CON*H*N); 8.18 (s, 1H, =CH), 7.55~7.34 (m, 8H, Ar-H), 3.84 (s, 3H, NCH_3_), 2.60 (s, 3H, pyrazole-CH_3_), 2.53 (s, 3H, pyrazole-CH_3_), 2.32 (s, 3H, Ph-CH_3_); ^13^C NMR (125 MHz, DMSO-*d_6_*): *δ* 164.2, 160.5, 148.9, 148.3, 140.4, 137.8, 136.5, 134.9, 133.1, 132.2, 129.8, 129.2, 127.9, 127.3, 126.6, 126.4, 126.3, 125.5, 125.3, 113.6, 113.0, 36.6, 19.1, 14.9, 14.1. IR (KBr, cm^-1^): ν3307.9, 3188.3, 3047.5, 2926.0, 1676.1, 1655.2, 1635.6, 1606.7, 1608.6, 1521.8, 1500.6, 1458.2, 1375.3, 1354.0.

**5-chloro-*N*-(2-(2-(furan-2-ylmethylene) hydrazinecarbonyl)-6-methylphenyl)-1, 3 -dimethyl-1*H*-pyrazole-4-carboxamide (7i)**

White solid, M.p. >250 ℃. ^1^H NMR (500 Hz, DMSO-*d_6_*, ppm): *δ* 11.76 (s, 1H, -CON*H*Ar), 9.55 (s, 1H, -CON*H*N)，8.19 (s, 1H, =CH), 7.85 (s, 1H, 5-furan-H ), 7.47 (t, 2H, *J*= 6.30 Hz, 4,6-Ph-H ), 7.34 (t, 1H, *J*= 7.73Hz, 5-Ph-H ), 6.92 (d, 1H, *J*= 3.4 Hz, 4- furan-H, ), 6.64 (t, 1H, *J*= 2.88 Hz, 4- furan-H ), 3.77 (s, 3H, NCH_3_), 2.32 (s, 3H, pyrazole-CH_3_), 2.26 (s, 3H, Ph-CH_3_); ^13^C NMR (125 MHz, DMSO-*d_6_*): *δ* 164.5, 160.5, 149.9, 148.4, 145.8, 137.9, 136.5, 134.9, 133.2, 132.1, 127.3, 126.6, 126.3, 111.1, 112.8, 112.8, 36.6, 19.1, 14.1. IR (KBr, cm^-1^): ν 3282.8, 3223.1, 3066.8, 2965.6, 2945.7, 2936.8, 2854.6, 1654.9, 1638.2, 1558.3, 1543.1, 1506.4, 1465.9, 1458.2, 1419.6, 1344.2.

**5-chloro-1, 3-dimethyl-*N*-(2-methyl-6-(2-(2-methylpropylidene) hydrazine carbonyl) phenyl)-1*H*-pyrazole-4-carboxamide (7j)**

White solid, M.p. 241~243 °C. ^1^H NMR (500 Hz, DMSO-*d_6_*, ppm): *δ* 11.32 (s, 1H, -CON*H*Ar), 9.54(s, 1H, -CON*H*N)，7.54 (d, *J* = 5.15 Hz, 1H, =CH), 7.43 (d, *J* = 7.45 Hz, 1H, 6-Ph-H), 7.39 (d, *J* = 7.45 Hz, 1H, 4-Ph-H), 7.30 (t, *J* = 7.43 Hz, 1H, 5-Ph-H), 3.78 (s, 3H, NCH_3_), 2.48～2.44 (m, 1H, C*H*(CH_3_)_2_), 2.32 (s, 3H, pyrazole-CH_3_), 2.23 (S, 3H, Ph-CH_3_), 1.04 (d, *J* = 6.85 Hz, 6H, CH(C*H*_3_)_2_); ^13^C NMR (125 MHz, CDCl_3_): *δ* 164.3, 160.4, 157.2, 148.4, 136.4, 134.8, 133.0, 132.1, 127.3, 126.5, 126.2, 112.8, 36.6, 20.1, 19.9, 19.1, 14.2. IR (KBr, cm^-1^): *ν* 3259.7, 3199.9, 3066.6, 2960.7, 2929.9, 2868.2, 1653.0, 1633.7, 1595.1, 1558.5, 1512.2, 1465.9, 1363.7.

**5-chloro-*N*-(4-chloro-2-methyl-6-(2-((1-methyl-1*H*-pyrrol-2-yl) methylene) hydrazinecarbonyl) phenyl)-1, 3-dimethyl-1*H*-pyrazole-4-carboxamide (7k)**

White solid, M.p. >250 °C. ^1^H NMR (500 MHz, DMSO-*d_6_*, ppm): *δ* 11.54 (s, 1H, -CON*H*Ar), 9.59(s, 1H, -CON*H*N)，8.22(s, 1H, -N=*CH*), 7.56(s, 1H, 6-Ph-H), 7.52(s, 1H, 4-Ph-H ), 6.97 (s, 1H, 2-pyrrol-H), 6.49 (d, 1H, *J*= 2.30 Hz, 4-pyrrol-H), 6.10 (t, 1H, *J*= 3.15 Hz, 3-pyrrol-H), 3.83 (s, 3H, NCH_3_), 2.31 (s, 3H, pyrazole-CH_3_); 2.25(m, 6H, pyrrol-CH_3_, Ph-CH_3_); ^13^C-NMR (125 MHz, DMSO-*d_6_*): *δ* 162.5, 160.5, 148.4, 141.8, 139.01, 134.1, 133.8, 132.3, 130.6, 128.8, 127.4, 125.9, 116.0, 115.3, 112.8, 109.0, 36.6, 36.4, 18.9, 14.1. IR (KBr, cm^-1^): ν 3265.5, 3167.1, 3099.6, 2966.5, 2928.8, 2848.9, 1653.0, 1631.5, 1610.6, 1558.5, 1541.1, 1521.8, 1473.6, 1448.5.

**5-chloro-*N*-(2-(2-(4-chlorobenzylidene) hydrazinecarbonyl)-6-methylphenyl)-1, 3 -dimethyl-1*H*-pyrazole-4-carboxamide (7l)**

White solid, M.p. >250 °C. ^1^H NMR (500 MHz, CDCl_3_, ppm): *δ* 11.87 (s, 1H, -CON*H*Ar), 9.56(s, 1H, -CON*H*N)，8.31(s, 1H, -N=*CH*), 7.73~7.31 (m, 7H, Ar-H), 3.76 (s, 3H, NCH_3_), 2.32 (s, 3H, pyrazole-CH_3_), 2.27 (s, 3H, Ph-CH_3_); ^13^C-NMR (125 MHz, DMSO-*d_6_*): *δ* 164.7, 160.6, 148.4, 146.8, 136.5, 135.1, 134.9, 133.7, 134.7, 133.2, 132.1, 129.5, 129.2, 129.0, 127.3, 126.6, 126.3, 112.9, 39.7, 19.0, 14.1. IR (KBr, cm^-1^): ν3255.8, 3186.4, 3049.5, 2929.9, 2848.9, 1651.1, 1637.6, 1597.1, 1552.7, 1510.6, 1402.3, 1303.9.

**5-chloro-*N*-(4-chloro-2-(2-((dimethylamino) methylene) hydrazinecarbonyl) -6-methylphenyl)-1, 3-dimethyl-1*H*-pyrazole-4-carboxamide (7m)**

White solid, M.p. 247~249 °C. ^1^H NMR (500 MHz, DMSO-*d_6_* ppm): *δ* 9.83 (s, 1H, -CON*H*Ar), 9.66(s, 1H, -CON*H*N)，7.73(s, 1H, -N=*CH*), 7.29(s, 1H, Ar-H), 7.21(s, 1H, Ar-H), 3.80 (s, 3H, NCH_3_), 2.85 (s, 6H, N(CH_3_)_2_), 2.50 (s, 3H, pyrazole-CH_3_), 2.23(s, 3H, Ph-CH_3_); ^13^C-NMR (125 MHz, CDCl_3_): *δ* 162.9, 160.9, 155.7, 150.6, 137.8, 133.6, 132.6, 130.8, 130.4, 128.6, 125.0,111.5, 36.4, 36.3, 19.5, 18.4, 14.7. IR (KBr, cm^-1^): ν 3252.0, 3203.8, 3161.3, 2976.2, 2953.0, 1647.2, 1597.1, 1577.8, 1533.4, 1450.5, 1404.2, 1377.1.

**5-chloro-N-(4-chloro-2-methyl-6-(2-(pyridin-2-ylmethylene)hydrazinecarbonyl)phenyl)-1,3-dimethyl-1H-pyrazole-4-carboxamide (7n)**

White solid, M.p. 235~237 °C. ^1^H NMR (500 MHz, DMSO-*d_6_*, ppm): *δ* 12.10 (s, 1H, -CON*H*Ar), 9.56(s, 1H, -CON*H*N)，8.62~ 8.61(d, 1H, *J*=5.15, 6-pyridine-H), 8.35(s, 1H, -N=*CH*), 7.96 (d, 1H, *J*=7.45, 6-Ph-H ), 7.90 (t, 1H, *J*=7.73, 4-pyridine-H ), 7.60 (d, 2H, *J*=6.30, 5-pyridine-H, 4-Ph-H ), 7.43~7.40 (m, 1H, 3-pyridine-H), 3.77 (s, 3H, NCH_3_), 2.32 (s, 3H, pyrazole-CH_3_), 2.29 (s, 3H, Ph-CH_3_); ^13^C-NMR (125 MHz, DMSO-*d_6_*): *δ* 163.3, 160.8, 153.8 , 150.1, 149.8, 148.8, 148.3, 139.1, 137.4, 133.9, 132.6, 130.8, 127.3, 126.0, 125.0, 120.4, 112.9, 36.5, 18.8, 14.0. IR (KBr, cm^-1^): ν 3369.6, 3244.3, 3184.5, 3047.5, 3005.2, 2933.7, 2858.5, 1660.7, 1637.6, 1560.4, 1521.8, 1491.0, 1471.7, 1435.0, 1375.3.

**5-chloro-*N*-(4-chloro-2-methyl-6-(2-(quinolin-2-ylmethylene)hydrazinecarbonyl)phenyl)-1,3-dimethyl-1*H*-pyrazole-4-carboxamide (7o)**

White solid, M.p. 235~237 °C. ^1^H NMR (500 MHz, DMSO-*d_6_*, ppm): *δ* 12.24 (s, 1H, -CON*H*Ar), 9.56(s, 1H, -CON*H*N)，8.47,(s, 1H, -N=*CH*), 8.43 (d, 1H, *J*= 9.15 Hz 4-quinoline-H), 8.10 (d, 1H, *J*= 8.60 Hz 5-quinoline-H), 8.05 (d, 1H, *J*= 8.55 Hz 8-quinoline-H), 8.01 (d, 1H, *J*= 8.08 Hz 3-quinoline-H), 7.81 (t, 1H, *J*= 8.55 Hz 7-quinoline-H), 7.65~7.60 (m, 3H, 6-quinoline-H, 6-Ph-H, 4-Ph-H), 3.74 (s, 3H, NCH_3_), 2.31 (s, 3H, pyrazole-CH_3_), 2.29 (s, 3H, Ph-CH_3_); ^13^C NMR (125 MHz, DMSO-*d_6_*): *δ* 163.4, 160.8, 154.2, 148.6, 148.2, 147.9, 139.2, 137.4, 134.1, 134.0, 132.6, 130.8, 130.7, 129.5, 128.6, 127.9, 127.3, 126.0, 118.0, 111.4, 113.0, 36.5, 18.8, 14.0. IR (KBr, cm^-1^): ν 3369.6, 3244.3, 3203.8, 3167.1, 3001.2, 2931.8, 2879.7, 1658.8, 1635.6, 1566.2, 1558.5, 1521.8, 1492.9, 1429.3, 1375.3.

**5-chloro-*N*-(4-chloro-2-(2-(2-fluorobenzylidene)hydrazinecarbonyl)-6-methylphenyl)-1,3-dimethyl-1*H*-pyrazole-4-carboxamide (7p)**

White solid, M.p. 243~245 °C. ^1^H NMR (500 MHz, DMSO-*d_6_*, ppm): *δ* 11.99 (s, 1H, -CON*H*Ar), 9.56(s, 1H, -CON*H*N)，8.55(s, 1H, -N=*CH*), 7.92~7.28 (m, 6H, Ar-H), 3.76 (s, 3H, NCH_3_), 2.31 (s, 3H, pyrazole-CH_3_), 2.27 (s, 3H, Ph-CH_3_); ^13^C-NMR (125 MHz, DMSO-*d_6_*): *δ* 163.1, 160.7, 160.3,148.3, 141.3, 139.1, 134.0, 133.8, 133.7, 132.5, 130.7, 127.3, 126.0, 125.5, 122.2, 116.7, 116.5, 112.9, 36.6, 18.8, 14.0. IR (KBr, cm^-1^): ν 3371.6, 2993.5, 2927.9, 2854.7, 1660.7, 1635.5, 1591.1, 1564.2, 1533.4, 1489.1, 1458.2, 1356.0.

**5-chloro-*N*-(4-chloro-2-methyl-6-(2-(4-methylbenzylidene)hydrazinecarbonyl) phenyl)-1,3-dimethyl-1*H*-pyrazole-4-carboxamide (7q)**

White solid, M.p. 247~249 °C. ^1^H NMR (500 MHz, DMSO-*d_6_*, ppm): *δ* 11.82 (s, 1H, -CON*H*Ar), 9.57(s, 1H, -CON*H*N)，8.27(s, 1H, -N=*CH*), 7.60~7.25 (m, 6H, Ar-H), 3.76 (s, 3H, NCH_3_), 2.34~2.25 (m, 9H, pyrazole-CH_3_, Ph- CH_3_); ^13^C-NMR (125 MHz, DMSO-*d_6_*): *δ* 163.0, 160.6,148.6, 148.3, 140.6, 139.1, 134.0, 133.8, 132.4, 131.9, 130.7, 129.8, 127.6, 127.4, 127.6, 127.4, 126.0, 112.8, 36.6, 21.6, 18.8, 14.0. IR (KBr, cm^-1^): ν 3250.1, 3194.1, 3053.3, 3012.9, 2918.3, 2854.7, 1651.1, 1623.5, 1558.5, 1508.3, 1461.5, 1363.7.

**5-chloro-*N*-(4-chloro-2-(2-(3-chlorobenzylidene)hydrazinecarbonyl)-6-methylphenyl)-1,3-dimethyl-1*H*-pyrazole-4-carboxamide (7r)**

White solid, M.p. >250 °C. ^1^H NMR (500 MHz, DMSO-*d_6_*, ppm): *δ* 12.02 (s, 1H, -CON*H*Ar), 9.54(s, 1H, -CON*H*N)，8.28(s, 1H, -N=*CH*), 7.76(s, 1H, 2-Ph-H), 7.67~7.48 (m, 5H, Ar-H), 3.76 (s, 3H, NCH_3_), 2.30 (s, 3H, pyrazole-CH_3_), 2.27 (s, 3H, Ph-CH_3_); ^13^C-NMR (125 MHz, DMSO-*d_6_*): *δ* 163.2, 160.7, 148.3, 146.7, 139.1, 136.9, 134.2, 134.0, 133.9, 132.5, 131.3, 130.7,130.4, 127.3, 126.8, 126.4, 126.0, 112.9, 36.6, 18.8, 14.0. IR (KBr, cm^-1^): ν 3367.7, 3184.5, 3151.7, 3014.7, 1660.7, 1647.2, 1560.4, 1554.2, 1539.6, 1490.1, 1465.1, 1357.9.

**5-chloro-*N*-(4-chloro-2-(2-(4-chloro-3-nitrobenzylidene)hydrazinecarbonyl)-6- methylphenyl)-1,3-dimethyl-1*H*-pyrazole-4-carboxamide (7s)**

White solid, M.p. >250 °C. ^1^H NMR (500 MHz, DMSO-*d_6_*, ppm): *δ* 12.19 (s, 1H, -CON*H*Ar), 9.53(s, 1H, -CON*H*N), 8.38~7.55 (m, 6H, Ar-H+ N=*CH* ), 3.76 (s, 3H, NCH_3_), 2.31 (s, 3H, Ph-CH_3_), 2.28 (s, 3H, pyrazole-CH_3_). ^13^C-NMR (125 MHz, DMSO-*d_6_*): *δ* 163.3, 160.8, 148.4, 148.3, 144.9, 139.1, 135.4, 134.00, 133.8, 132.8, 132.6, 132.1, 130.7, 127.3, 126.4, 126.0, 124.1,112.9, 36.5, 18.8, 14.0. IR (KBr, cm^-1^): *ν* 3371.6, 3180.6, 3016.7, 2929.9, 2854.7, 1660.7, 1637.6, 1610.6, 1560.4, 1537.3, 1521.8, 1491.0, 1346.3.

# References

1. Park HJ, Lee K, Park SJ, Ahn B, Lee JC, Cho HY, Lee KI: **Identiﬁcation of antitumor activity of pyrazole oxime ethers**. *Bioorg. Med. Chem. Lett.* 2005. **15**: 3307-3312.
2. John L, Huppatz: **Systemic Fungicides. The Synthesis of Certain Pyrazole Analogues of Carboxin**. *Aust. J. Chem*. 1983, **36**: 135-147.
3. Mohareb RM, Aziz SI, Abdel-Sayed NI, El-Ablack FZ: Reactions of Benzoylisothiocyanate with Acetoacetanilide: **Synthesis of Pyrazole, Pyridine, Pyrimidine, Pyrazolo [3, 4-*d*] pyrimidine, Pyrazolo [4, 3-*d*] pyrimidine and Pyrido [4, 3-*d*] oxazine Derivatives**. *Collect. Czech. Chem. Commun.* 1993, **58**: 947-953.
4. Du HT, Ou-Yang GP, Du HJ, Sh DB, Jin LH: S**ynthesis and Biological Activity of N-(Substituted-*p*-trifluoromethyl-phenyl)-1, 3, 5-trisubstituted-pyrazole-4- carboxamie Derivatives**. *Chin. J. Org. Chem.* 2006, **26**: 383-386. (in chinese)

# Copies of ^1^H-NMR and ^13^C-NMR of 7a-s


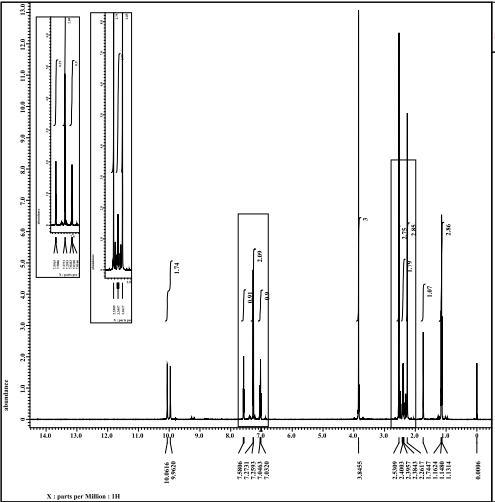


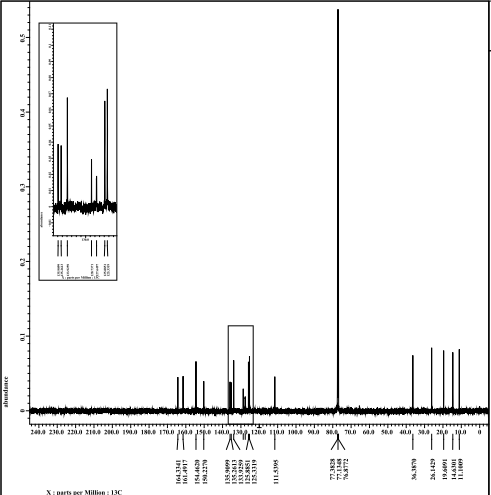


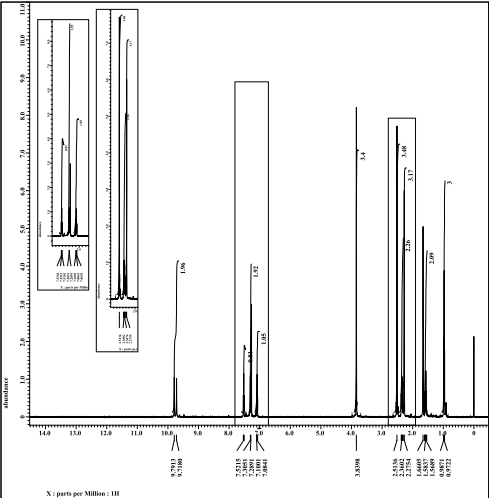


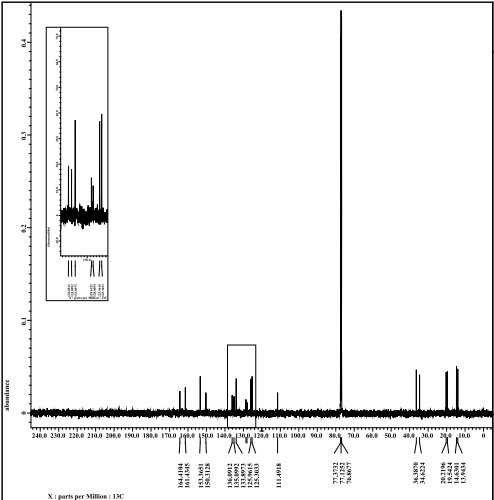


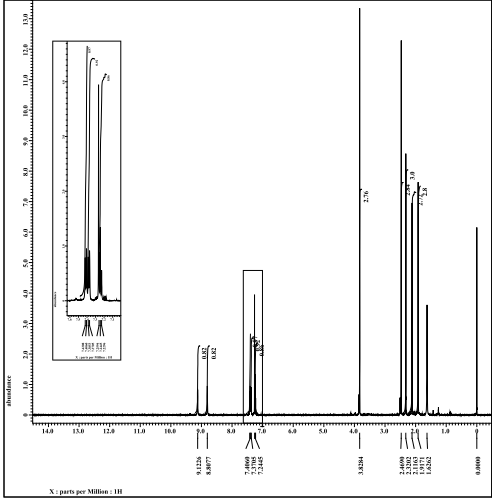


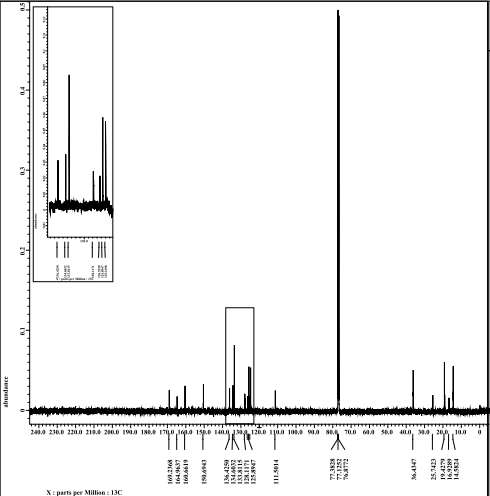


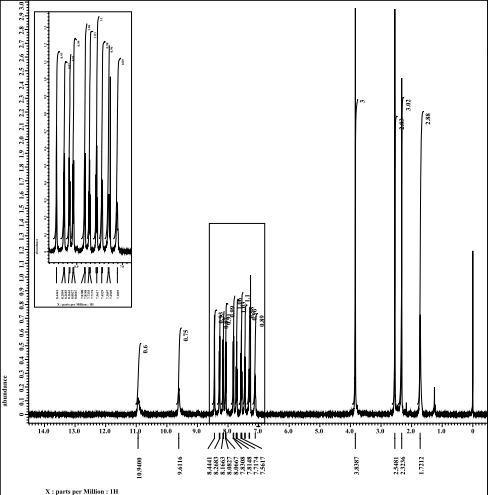


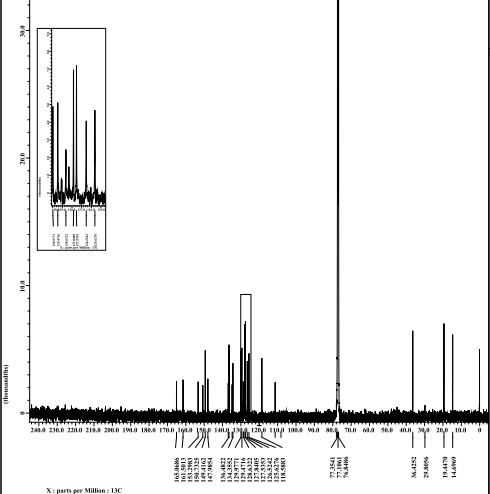


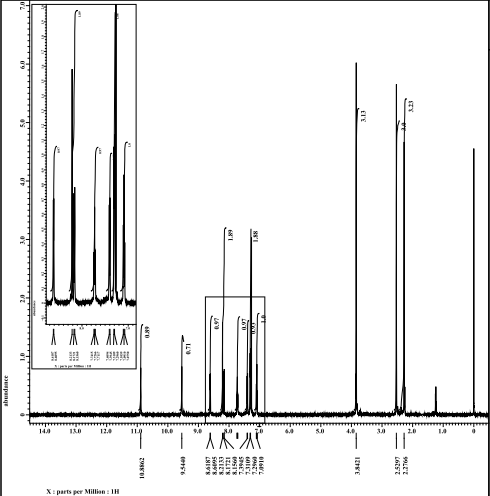


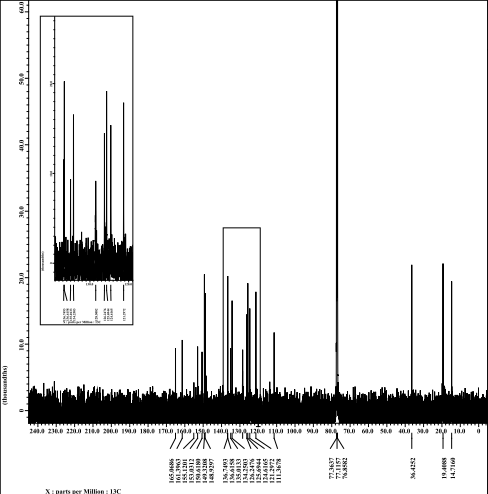


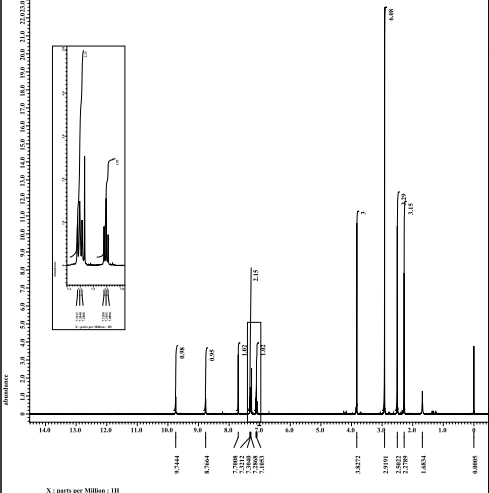


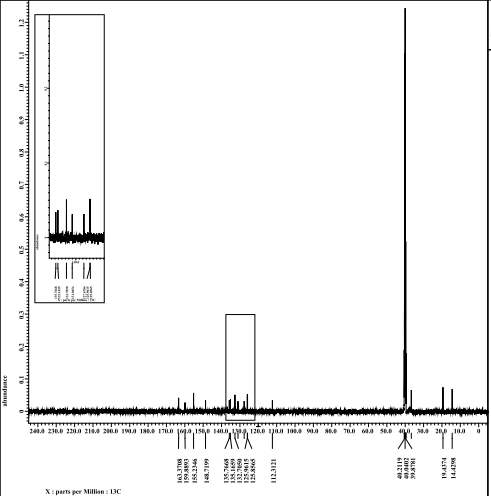


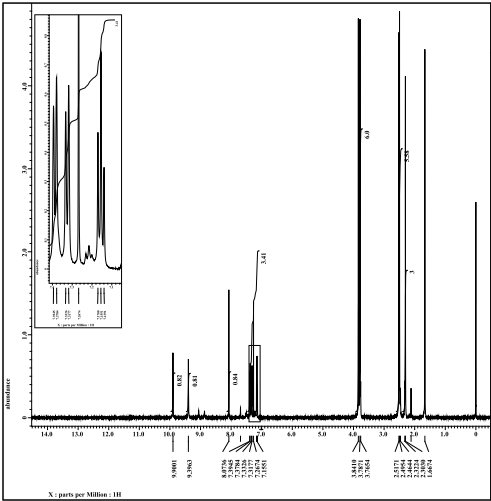


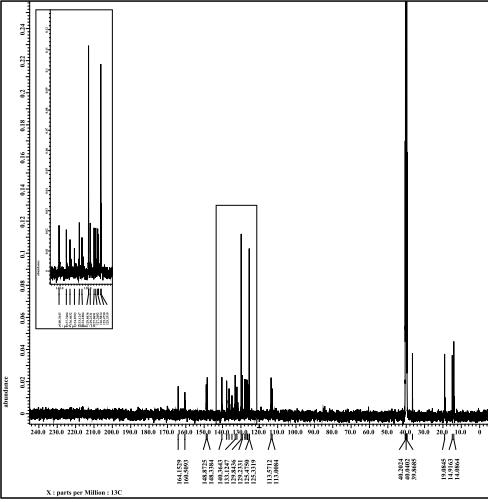


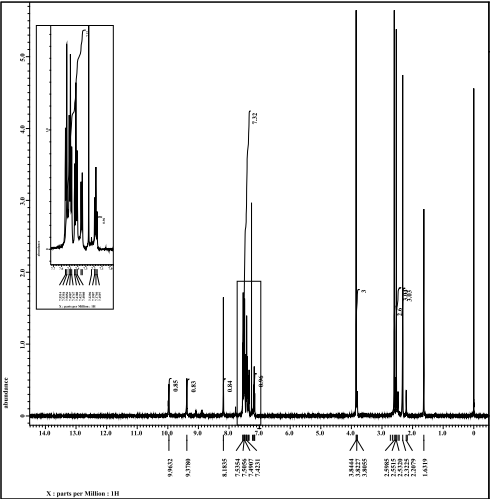


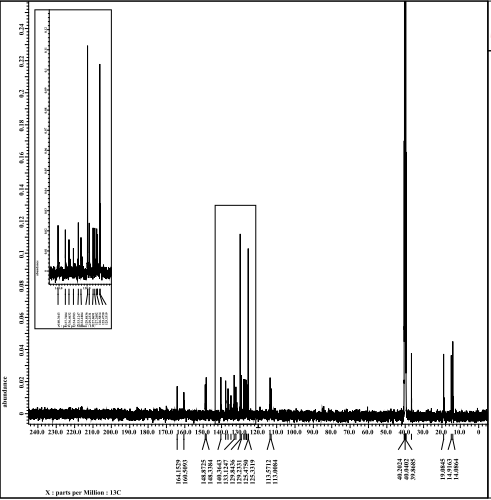


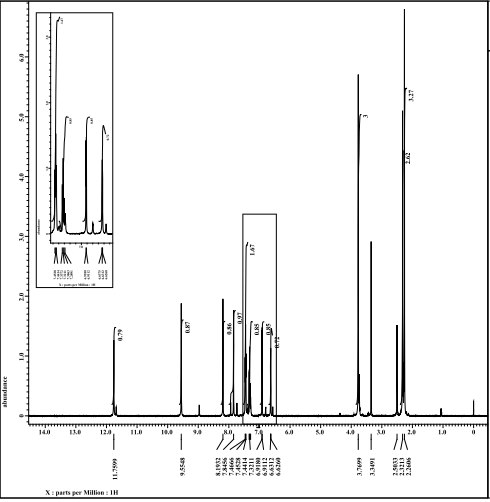


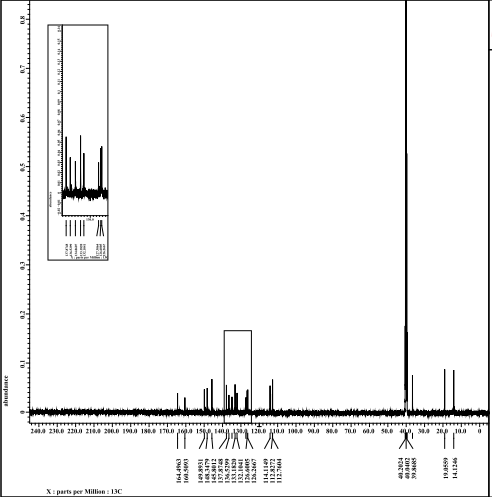


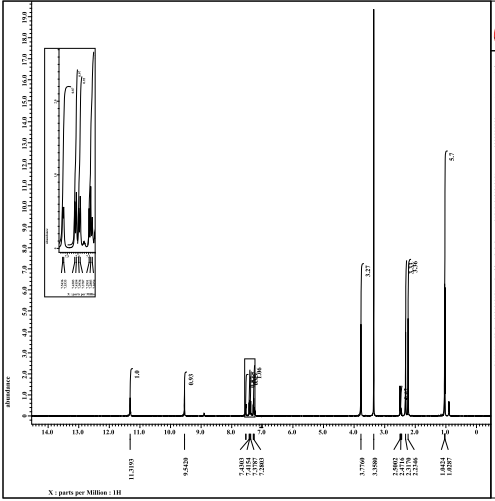


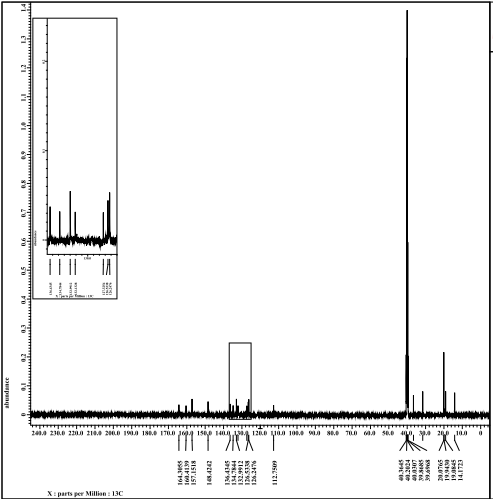


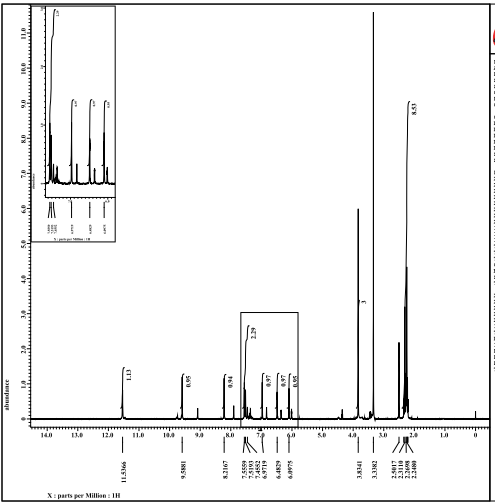


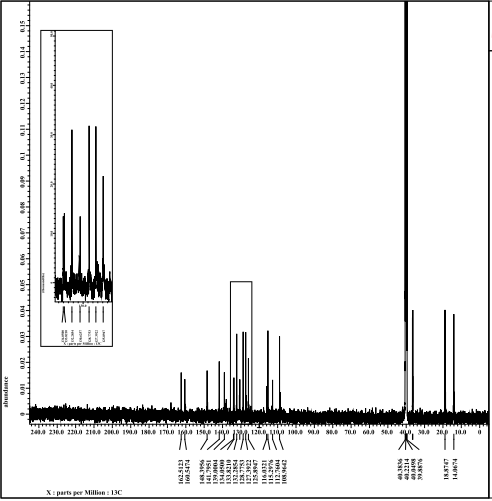


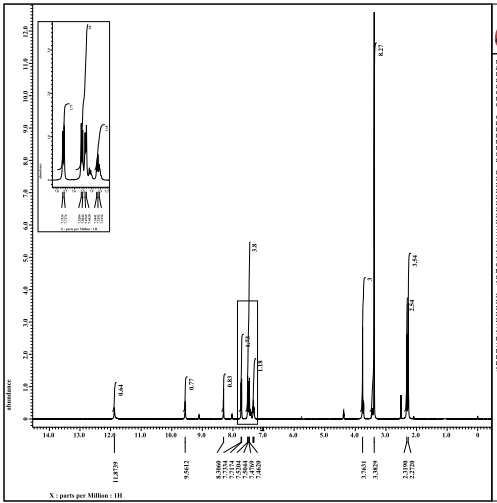


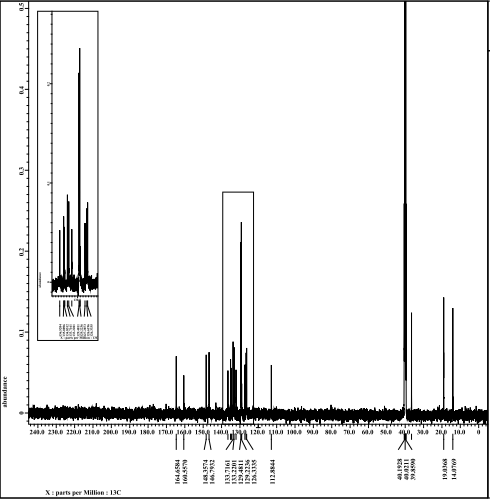


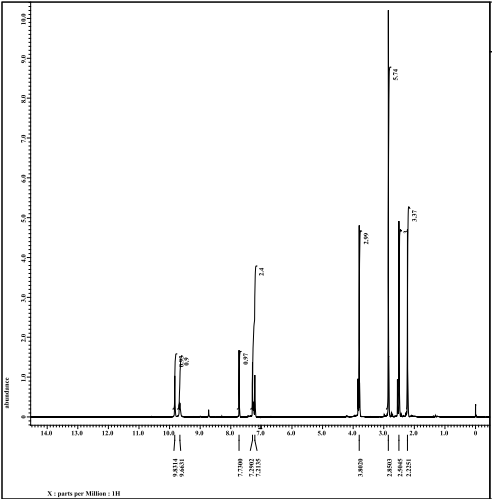


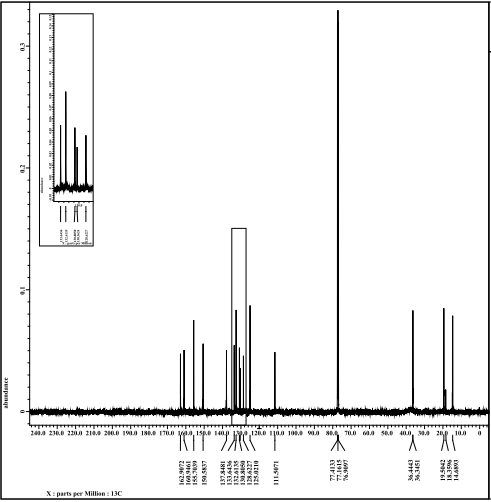


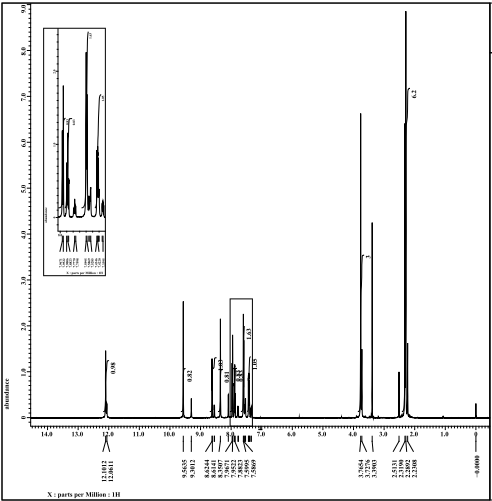


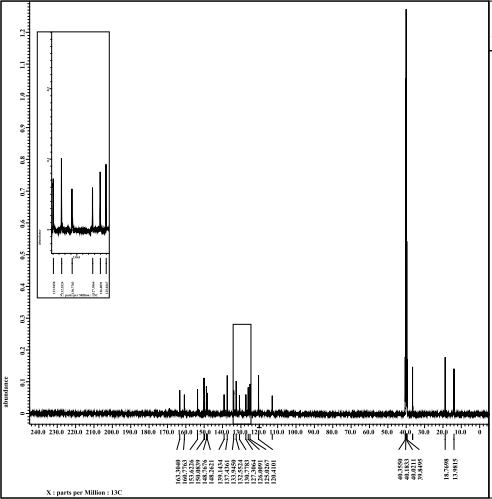


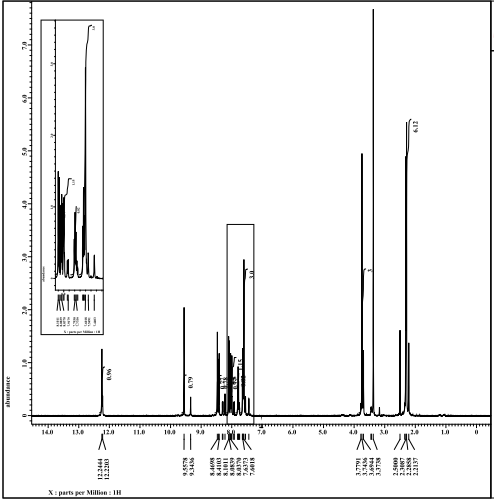


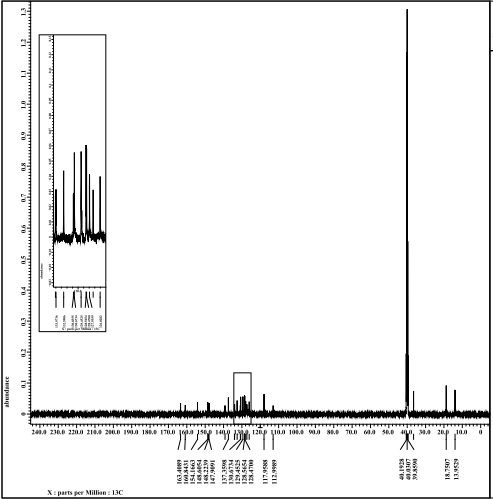


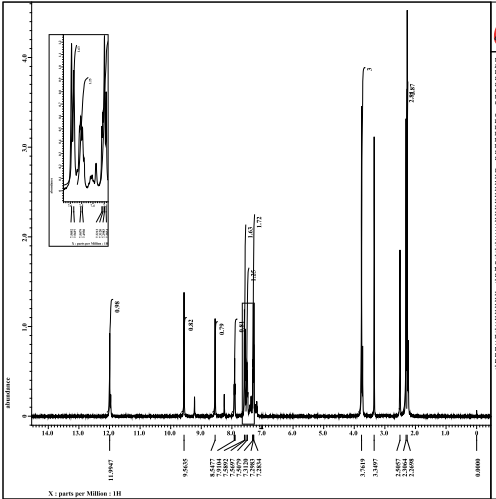


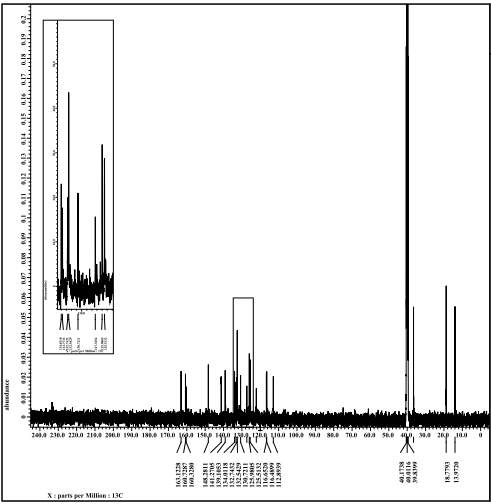


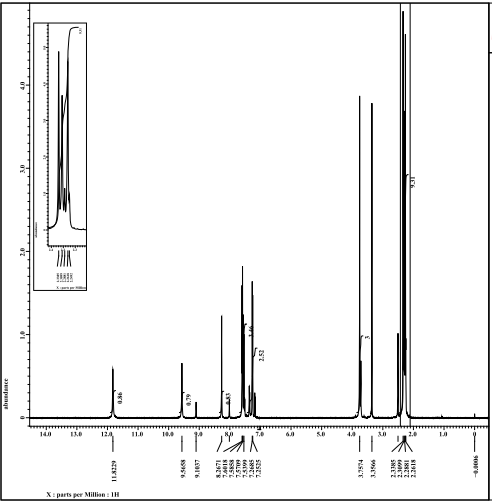


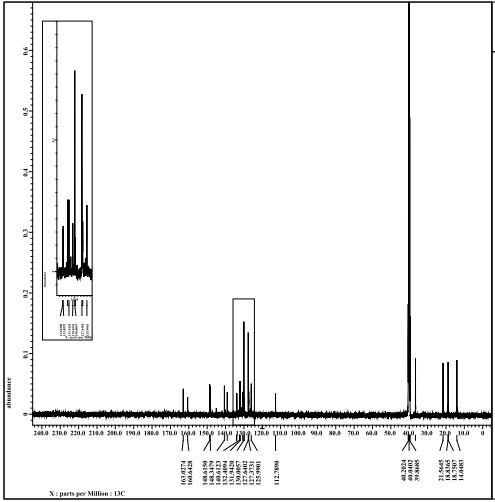


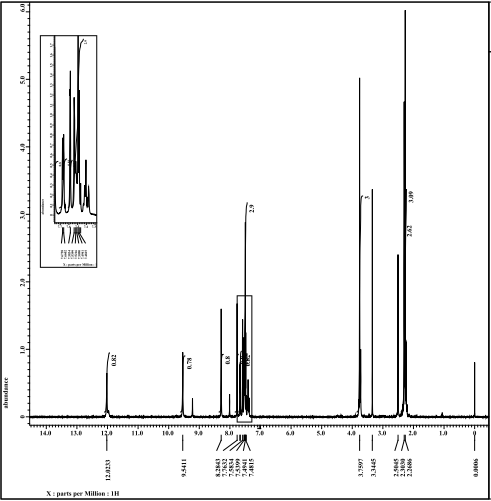


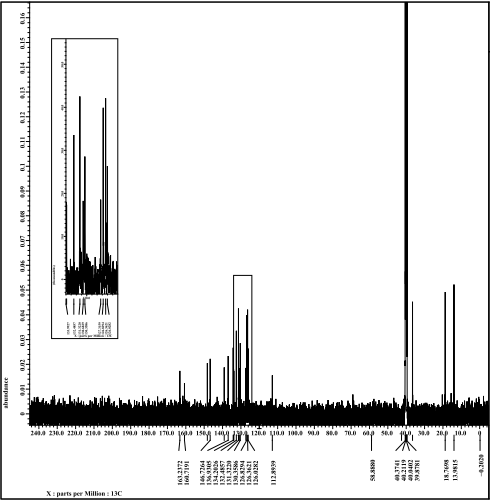


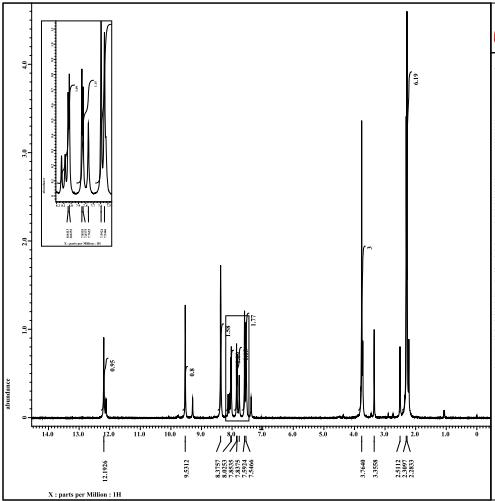


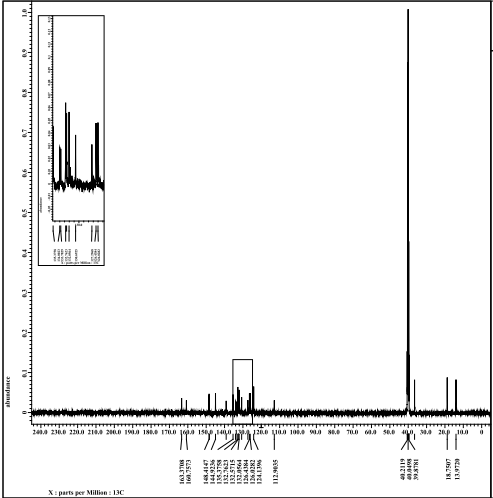

Supplement: Additional file 3 — Experimental details and data of title compounds 7a-s. Which includes the experimental procedure, spectroscopic data of intermediate 5, intermediate 6, title compounds 7a-s, copies of 1H NMR and 13C NMR. [file 1752-153X-6-51-S3.doc]
